# Effectiveness of intensive healthcare waste management training model among health professionals at teaching hospitals of Pakistan: a quasi-experimental study

**DOI:** 10.1186/s12913-015-0758-7

**Published:** 2015-02-28

**Authors:** Ramesh Kumar, Ratana Somrongthong, Babar Tasneem Shaikh

**Affiliations:** Fellow at College of Public Health Sciences, Chulalongkorn University Thailand, Bangkok, Thailand; Department of Health Systems and Policy, Health Services Academy, Islamabad, Pakistan; College of Public Health Sciences, Chulalongkorn University, Bangkok, Thailand

**Keywords:** Health care workers, Waste management, Infectious waste, KAP, Quasi-experimental study

## Abstract

**Background:**

Infectious waste management has always remained a neglected public health problem in the developing countries, resulting in high burden of environmental pollution affecting general masses. Health workers are the key personnel who are responsible for the management of infectious waste at any hospital, however**,** their proper training and education is must for an optimal performance. This interventional study was conducted to assess the effectiveness of Intensive healthcare waste management (IHWM) training model at two tertiary care hospitals of Rawalpindi city, Pakistan.

**Methods:**

This study was quasi-experimental pre and post design with control and intervention groups. Out of 275 health care workers enrolled for the study, 138 workers were assigned for intervention group for 3 months trainings, hands-on practicum and reminders on infectious waste management; whereas 137 workers were assigned to the control hospital where routine activities on infectious health care waste management were performed. Pre and post intervention assessment was done for knowledge, attitude and practices (KAP); and was statistically analyzed. Bivariate and multivariate analysis, independent, paired and unpaired *t*-test, chi-square with p values, and mean of the responses were calculated. Overall the response rate was 92% at the end of intervention.

**Results:**

During the baseline survey, 275 healthcare workers (HCW) included doctors, nurses, paramedics and sanitary workers, and after 3 months of intervention, 255 were reached out to complete the questionnaire. With regard to KAP at baseline, there were no significant differences between two groups at baseline, except for gender and department. However, in the post intervention survey, statistically significance difference (<0.05) between intervention and control group’s knowledge, attitude and practices was found. Moreover, within the control group no statistically significant difference was reported (>0.05) after 3 months.

**Conclusions:**

Study results suggest that IHWM training could be an effective intervention for improving knowledge, attitudes and practices among health workers regarding infectious waste management. Such training should become a regular feature of all hospitals for reducing the hazards attached with infectious wastes.

## Background

Across the world, tertiary hospitals face huge challenge of ensuring an infection free environment for their clients [1]. Compounding the situation are the poor infectious waste management practices among health care workers in the hospitals, which pose a big public health threat [2]. Improper management of infectious waste frequently infects the health workers with Hepatitis B & C, typhoid, cholera, tuberculosis, skin infections, respiratory infections and HIV/AIDS [3,4]. The infectious healthcare waste is considered to be the second most risky waste in the world that should be handled properly by the trained staff within an organization. Infectious waste is produced during the patient care at hospitals, clinics, maternity homes and research institutes [5]. Medical waste produced by each bed across the world ranges from 0.5-2 kg/day [6].

Quantity of infectious hospital waste produced in Pakistan is not different from the global figures [7]. Proper periodic training is therefore needed for to improve the knowledge, attitude and practices of health workers who are handling the waste in routine [2]. Scavengers and waste handlers with low socio economic groups are more involved in the recycling of used syringes at developing countries and get frequent needle prick injuries [8]. These poor practices of waste management are reported in India, China and Bangladesh too, resulting in environmental threats to the populations as well as major occupational risk [9].

Studies also suggested that the practices of health care workers are not up to the standards which lead to major threats of environmental pollution. Segregation is the main step which is not being practiced in the hospitals by health staff. One possible reason is lack of training [10]. A recent study has also reported that the waste management practices even among general practitioners were not appropriate, hence this group too needs to be trained [11]. Support from hospital administration is critical for conducting the regular training and refresher courses for staff handling infectious wastes [7,12]. Most of the waste produced from the hospital is non-infectious which can easily be managed through local municipality; but 10-15% infectious waste needs special attention by the trained health staff, otherwise this small proportion may contaminate the entire lot. This hazard can only be averted if waste handling staff is trained in segregation technique at point source [13].

Hitherto, there has been no study to assess the effectiveness of infectious waste management training in Pakistan. Our interventional study was conducted through implementation of a new training model comprising deductive training, hands-on practicum and a reminder service in a tertiary care hospital with the objective to assess its effects on knowledge, attitude and practices of health care workers vis-à-vis infectious waste management in tertiary hospitals in Pakistan.

## Methods

**Figure 1:**
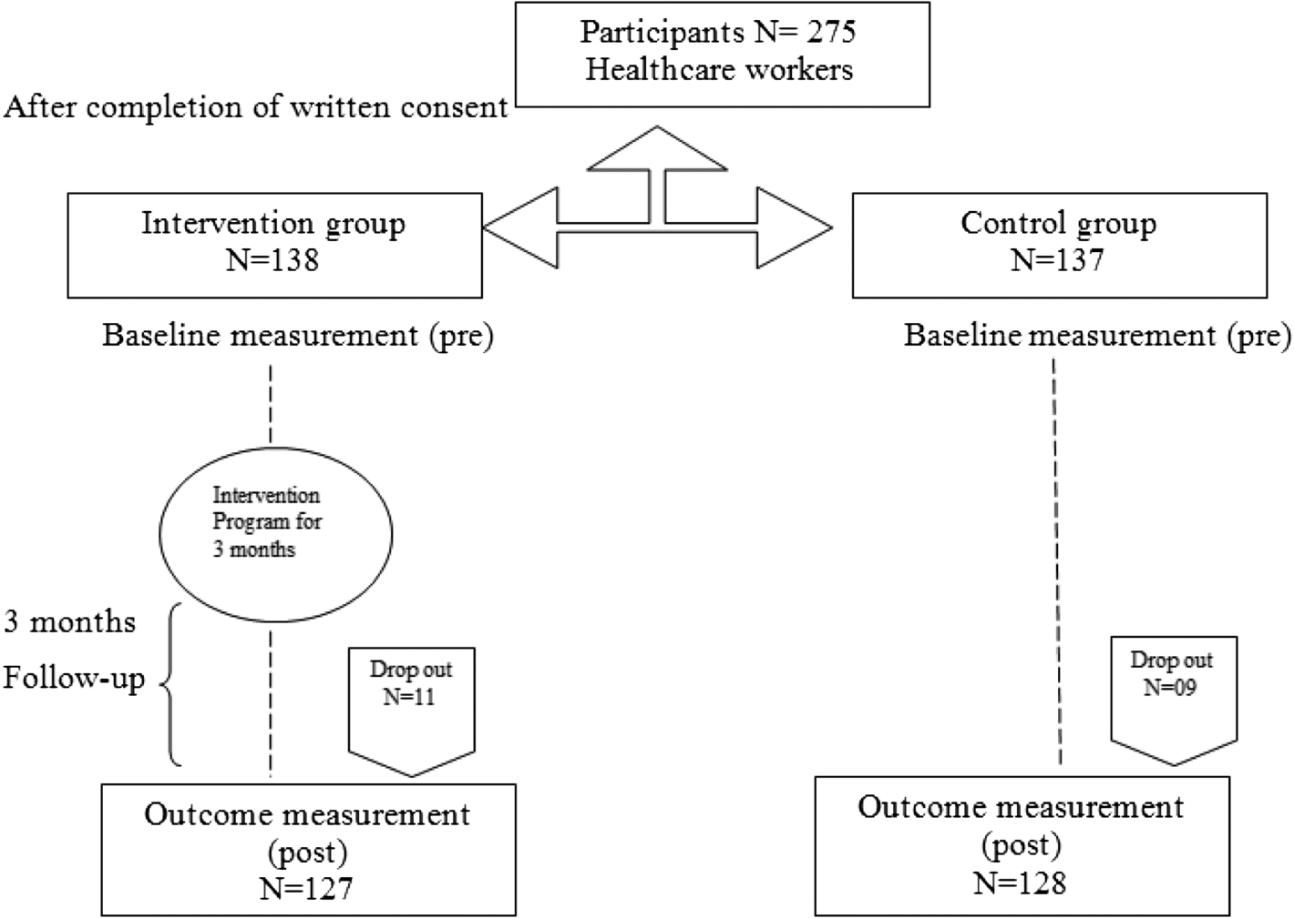
**Flow chart for quasi-experimental study.**

This was a quasi-experimental with control and intervention design conducted at two teaching hospitals located at Rawalpindi city-Pakistan from October 2013 to March 2014. Pre and post measurements were made through the World Health Organization (WHO) tool which was modified, pretested, piloted on 30 HCWs in adjacent district with similar kind of hospital before the study [14]. The internal consistency of the questionnaires was measured through Cronbach alpha for attitude and practice (0.92) and Kuder-Richardson (K-R 20) for knowledge (0.81) [15]. Sample size were calculated by using the 80% power, 0.05 alpha with 20% difference, and 275 health professionals including doctors, nurses, paramedics and sanitary workers were selected for this interventional study. These HCWs were selected randomly from employees list obtained from administration of both the hospitals and were invited for voluntary participation in the study. Hence, 138 HCWs for intervention and 137 HCWs were included in the control hospital, after obtaining written consent. The intervention and control hospital both are government tertiary healthcare facilities, therefore, using pick from hat method, one was labeled as intervention and other as control site. All HCWs were regular employees; and therefore students, house officers and trainee doctors were not included. 20 HCWs were dropped out due their transfer, causal leave or refusal (Figure 1).

Data were collected through self-administered questionnaire from doctors, nurses and paramedics, however, this instrument translated into local language to make it understandable even for the sanitary staff with low or no schooling, ensuring that they can read and understand the questions. Data collectors were selected from the other city and they had no affiliation with the study hospitals. They were trained before the study by the principal investigator. Research Ethics Committee of Health Services Academy, Pakistan (F.No.3-107/2013-IERC/HSA) approved the study; while institutional permission was also taken from the heads of both hospitals.

### Statistical analysis

SPSS version 22 was used for data analysis during this study [16]. The statistical analyses were performed to see the effect of the several individual characteristics on the outcomes of interest (knowledge, attitude and practices) by using appropriate bivariate analysis such as chi-square test for categorical variables and *t*-test for continuous variables. Paired and unpaired *t* test were used to see the effectiveness of training model with and within the groups. Then, multivariate linear and logistic regression was performed. Main outcome of interest was improved practices about infectious waste management (no = 0, yes = 1), knowledge about segregation, collection, storage and disposal (no = 0, yes = 1), and the attitude was measured through 5 point Likert’s scale. There were 24 statements for knowledge, 12 for attitude and 20 for practices; their mean score of responses were calculated and finally grouped in different levels. Baseline was conducted before the intervention and endline was conducted after 3 months period. Three months were allocated for intervention period.

### IHWM training model

**Figure 2:**
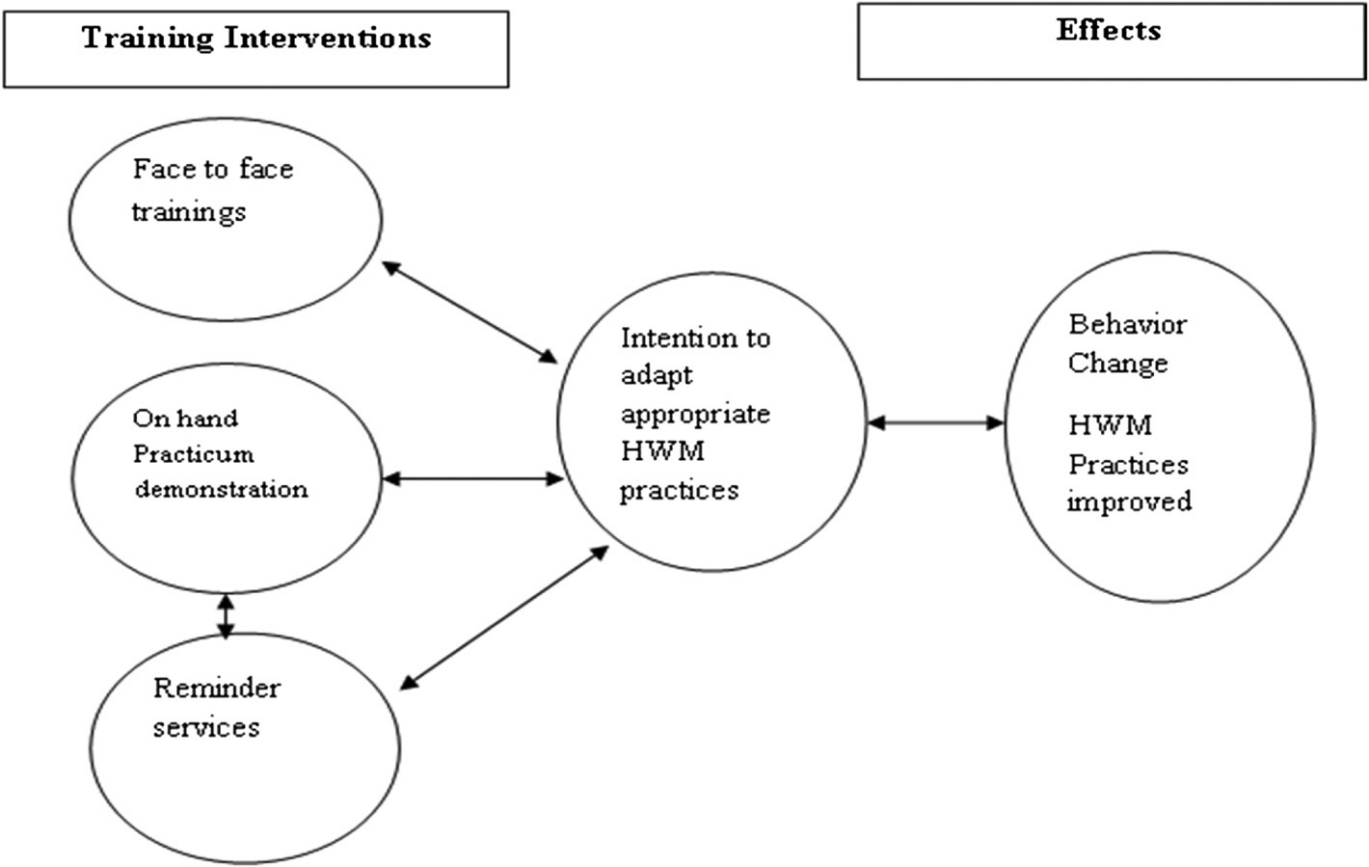
**Effective training intervention theory.**

IHWM training model (Figure 2) was based on the literature review mainly of WHO, and modified from the previous training models based on behavioral change theories [17].

The frequency of routine work and improved quality of practice regarding the infectious waste management were expected outcomes of this training. We implemented this training model for three months in the intervention hospital only; while the regular activities went on in the control hospital. Three training sessions each with 6 hours contact were conducted for HCWs in the intervention arm with four week interval between the two training. Training was followed by hands-on practicum demonstration for 6 hours duration each on the use of Personal Protective Equipment (PPE) and different steps of infectious waste management. PPE were also provided during the study to see the proper effectiveness of this training model. Third approach of this training model was the reminder services on infectious waste management which were given through administrators of the concerned departments during weekly morning meeting for three months. Trainings modules were adopted from WHO manual and guidelines on hospital waste management and hospital waste management rules 2005 [14,18,19].

## Results

Total 275 subjects were included in the baseline and final 255 have completed the intervention (92%) and were included in the final data analysis at endline. About the training, participants enjoyed the whole experience and shared their positive comments about the objectives, content, approach, material, ambiance and quality of instruction. Table 1 presents the socio-demographic characteristics of health care workers in both hospitals. Nearly half (47%) of the participants in intervention group and (42%) in the control group belong to age < 25 years. About gender distribution, the M:F ratio was 47:53 in intervention group, and 66:34 in the control group. More than one third in control as well as intervention group had graduation level of education. Almost half (49%) of the subjects from the intervention hospital were working in medicine department; while 37% were working in emergency and operation theaters in control group. Majority (54%) of the HCWs in control group and 47% of the HCWs in intervention group had working experience of less than 5 years. HCWs were assigned in two groups, health staff 81% in intervention and 80% in control hospital which includes doctors, nurses and paramedics; while the other group comprised of sanitary workers-19% in intervention and 20% in control. Age, educational status, income, experience and cadre had no significant association with KAP in both groups; while gender and affiliated department had statistically significance association with KAP in both the groups (p < 0.05).

**Table 1:** **Socio-demographic characteristics of the participants (n = 275)**

**Independent variables**		**Groups**						
		**Intervention**		**Control**		**Total**		**p value**
		**N**	**%**	**N**	**%**	**N**	**%**	
**Age**	<25	65	47%	58	42%	123	45%	0.706
	26-35	29	21%	33	24%	62	23%	
	>36	44	32%	46	34%	90	32%	
**Gender**	Male	65	47%	90	66%	155	56%	0.002
	Female	73	53%	47	34%	120	44%	
**Educational status**	Post-graduation	11	8%	12	9%	23	8%	0.844
	Graduation	48	35%	51	37%	99	36%	
	Secondary	53	38%	50	36%	103	38%	
	Primary	26	19%	24	18%	50	18%	
**Income**	<10,000	33	24%	39	29%	72	26%	0.212
	10-20 K	33	24%	41	30%	74	27%	
	>20,000	72	52%	57	42%	129	47%	
**Department**	Medicine and allied	67	49%	33	24%	100	36%	0.000
	Surgery and allied	45	33%	46	34%	91	33%	
	Emergency & operation theater	20	14%	51	37%	71	26%	
	Administration	6	4%	7	5%	13	5%	
**Experience**	<5 years	47	34%	54	40%	101	37%	0.638
	5-10	50	36%	47	34%	97	35%	
	>10 years	41	30%	36	26%	77	28%	
**Profession**	Doctors	41	30%	39	29%	80	29%	0.996
	Paramedics	28	20%	28	20%	56	21%	
	Nurses	43	31%	43	31%	86	31%	
	Sanitary workers	26	19%	27	20%	53	19%	

The respondent’s knowledge about different steps of infectious waste is presented in Table 2. The analysis of the knowledge questions showed that the frequencies of the correct answers varied from 35% to 85%. Knowledge has increased in the intervention group after the successful training model intervention from 20-25%; while in the control group only 1-3% knowledge has improved. Table 3 shows that the HCWs in both intervention and control groups were tested by using independent sample *t* test and chi-square before the intervention to find any significant difference regarding knowledge, attitude and practices between the two groups. The score was added to find the mean score. However, there were no statistically significant differences found between the groups at the baseline, before to start the intervention (p >0.05). Table 4 shows the statistical change of knowledge, attitude and practices regarding infectious waste management before and after the intervention by comparing mean score within the groups. Mean score was calculated by adding up the score on correct response. Wilcoxon signed Rank test was used to determine the mean difference between the knowledge and practices of the HCWs before and after the intervention within the groups, there was a significant difference in knowledge, attitude and practices (p < 0.05) has been reported in intervention group. However, no statistically significant difference within control group before and after was reported. For attitude of the HCWs, paired sample *t*-test was used to determine the mean difference in attitude before and after the intervention. In the intervention group, there was a significant difference p < 0.05; however, in the control group there was no significant difference.

**Table 2:** **Percentage of Knowledge of subjects regarding infectious waste (pre & post)**

**#**	**Knowledge statement**	**Correct answers**			
		**Intervention group**		**Control group**	
**Health staff**		**Pre N = 112**	**Post N = 101**	**Pre N = 110**	**Post N = 102**
1	Segregation of infectious waste	46%	66%	43%	43%
2	Collection of infectious waste	55%	79%	54%	57%
3	Storage of infectious waste	59%	78%	59%	59%
4	Disposal of infectious waste	57%	85%	65%	57%
**Sanitary workers**		**Pre N = 26**	**Post N = 26**	**Pre N = 27**	**Post N = 26**
1	Segregation of infectious waste	36%	61%	35%	34%
2	Collection of infectious waste	45%	67%	41%	43%
3	Storage of infectious waste	40%	64%	37%	36%
4	Disposal of infectious waste	43%	68%	40%	39%

**Table 3:** **Mean differences of KAP between groups at baseline**

**Variables**	**Intervention group**			**Control group**				
**Health staff**	**Mean SD**	**95% CI Low up**		**Mean SD**	**95% CI Low up**		**Chi square**	**p value**
Knowledge	12.80 (3.28)	-882040	.91845	12.75 (3.29)	-82042	091847	.003	.999
Attitude	27.38 (7.63)	-1.94216	2.11002	27.30 (7.63)	-1.94227	2.11013	.022	.495
Practice	11.26 (4.04)	-1.01611	1.53365	11.00 (5.49)	-1.02026	1.53780	.011	.512
**Sanitary workers**								
Knowledge	8.30 (3.12)	-82987	2.26007	7.59 (2.45)	-83972	2.26992	1.11	.573
Attitude	27.80 (8.27)	-3.84722	5.90705	26.77 (8.95)	-3.84488	5.90471	.211	.478
Practice	2.50 (3.82)	-2.19243	2.00724	2.59 (3.78)	-2.19302	2.00784	.002	.680

**Table 4:** **Mean difference in Knowledge, attitude & practices scores within groups after the intervention**

**Variables**	**Intervention**			**Control**		
**Health staff**	Pre mean (SD)	Post mean (SD)	p value	Pre mean (SD)	Post mean (SD)	p value
Knowledge	12.80 (3.28)	18.59 (2.25)	0.000	12.75 (3.29)	12.88 (3.32)	0.932
Attitude	27.38 (7.63)	34.12 (4.17)	0.000	27.30 (7.63)	27.00 (7.54)	0.738
Practices	11.26 (4.04)	14.81 (2.50)	0.000	11.00 (5.49)	11.05 (5.58)	0.912
**Sanitary workers**						
Knowledge	8.30 (3.12)	12.96 (3.07)	0.000	7.59 (2.45)	7.50 (2.41)	0.47
Attitude	27.80 (8.27)	31.84 (4.91)	0.021	26.77 (8.95)	26.80 (9.12)	1.000
Practices	2.50 (3.82)	9.23 (3.03)	0.000	2.59 (3.78)	2.46 (3.7)	1.000

## Discussion

### Trainings on infectious waste

This interventional study is one its own kind of research which has meticulously dealt with HCW’s knowledge, attitudes, and practices towards infectious waste management. Training model has proved that a continuous education of professionals could improve their overall approach toward the infectious waste management. Other studies also suggested that such trainings are very important to improve the waste handling practices of the staff in hospital environment [7,20]. Improper waste handling could only be minimized through continuous education of workers at their duty stations [11]. Another study has reported that HCWs must need regular information and reinforcing messages on the management of infectious waste [21]. Performance of health workforce could be enhances by intensive and then periodic refresher training [22].

### Reminder services

One of the components of our training model was reminder service by hospital administration given during their weekly staff meeting on regular basis for three months that would encourage all the employees to improve their attitude at their working place. This newly developed component of reminder services was previously never tried during various operations researches on infectious waste management. Nevertheless, these reminder services have successfully proved to achieve health behavior modification [17]. Therefore, this study included the reminder service managed by the hospital administration as part of the intervention, with an expectation that it would help sustain good health waste management practices.

### Hands-on practicum

This has been observed that the usage of Personnel Protective Equipment (PPE) during the study through hands-on practical demonstration in intervention hospital has brought the positive results in improving the proper practice of waste handling. Hence, it means that the practices of staff invariably depend on the availability of the PPE such as, aprons, masks, rubber boots etc. There was no change in practices reported in the control hospital unquestionably because of the non-availability of these PPE. Individual trainings with demonstration are the most efficient approach to instruct and visualize the proper techniques to use PPE. Our study also showed that the workers lack proficiency in practicing the proper waste bin color coding and the use of PPE at their work place. This could be improved with the support of the hospital management and by allocating the proper budget for periodic trainings on all such aspects in a tertiary hospital [23]. It has also been reported that there is very small gain in mean of knowledge, attitude and practices with in the control group. The possible reason is evident: no training no practicum and no reminder service. Literature proved that the practical demonstration has positively influence the practices of an individual and their behavior at their work place [24]. Face to face trainings has been proven to be one of more effective strategies for improving the practices and health behavior, especially when combined with other training interventional approaches [25]. Some of the limitations of our study are that these findings may not be applied at every level of healthcare facility in the country, and results represent only tertiary care setting; and that too in a relatively short experience. Due to limited finances, we could not offer any monetary incentives to the study participants. As both the hospital were located at the same city there was a slight probability of contamination between the groups. However, the distance between both hospitals was more than 5 kilometers. Lastly, this intervention might not have benefited all the health care workers due to the nature and time constraints for the intervention.

## Conclusion

This study is just an entré into this field and evaluations performed over longer periods in multiple hospitals and at different levels of care would definitely yield even richer evidence. Statistical analyses of the knowledge, attitude and practices of the trained staff after a year or so will potentially present an impact inference of this training. Hospitals must plan periodic evaluations after this training to gauge the change in the behaviors too, once the changes in facilities are instituted for the effective waste management, as recommended by the study. The results of this study suggest that use of an IHWM training model could improve knowledge and attitudes in regulated medical waste management. Such improvement could translate into improved performance. Therefore, it is proposed that the health policy makers and hospital authorities must replicate this knowledge translation program in other hospitals of the country to manage the big menace because of ineffective and unprotected infectious waste handling.

## References

[1] Maltezou HC, Fusco FM, Schilling S, De Iaco G, Gottschalk R, Brodt HR (2012). Infection control practices in facilities for highly infectious diseases across Europe. J Hosp Infect.

[2] Kumar R, Samrongthong R, Shaikh BT (2013). Knowledge, attitude and practices of health staff regarding infectious waste handling of tertiary care health facilities at metropolitan city of Pakistan. J Ayub Med Coll Abbottabad.

[3] Qaiser S, Arif A, Quaid S, Ahsan T, Riaz K, Niaz S (2013). Innovative solution to sharp waste management in a tertiary care hospital in Karachi. Pakistan Infect Control Hosp Epidemiol.

[4] Zhang HJ, Zhang YH, Wang Y, Yang YH, Zhang J, Wang YL (2013). Investigation of medical waste management in Gansu Province. China Waste Manag Res.

[5] Marinkovic N, Vitale K, Janev Holcer N, Dzakula A, Pavic T (2008). Management of hazardous medical waste in Croatia. Waste Manag.

[6] Ferreira V, Teixeira MR (2010). Healthcare waste management practices and risk perceptions: findings from hospitals in the Algarve region. Portugal Waste Manag.

[7] Kumar R, Khan EA, Ahmed J, Khan Z, Magan M, Nousheen N (2010). Healthcare waste management in Pakistan: current situation and training options. J Ayub Med Coll Abbottabad.

[8] Nema A, Pathak A, Bajaj P, Singh H, Kumar S (2011). A case study: biomedical waste management practices at city hospital in Himachal Pradesh. Waste Manag Res.

[9] Harhay MO, Halpern SD, Harhay JS, Olliaro PL (2009). Health care waste management: a neglected and growing public health problem worldwide. Trop Med Int Health.

[10] Paudel R, Pradhan B (2010). Health care waste management practice in a hospital. J Nepal Health Res Counc.

[11] Qaiser S (2012). Survey of sharp waste disposal system in clinics of New Karachi. J Pak Med Assoc.

[12] Oroei M, Momeni M, Palenik CJ, Danaei M, Askarian M (2014). A qualitative study of the causes of improper segregation of infectious waste at Nemazee Hospital, Shiraz. Iran J Infect Public Health.

[13] Haylamicheal ID, Desalegne SA (2012). A review of legal framework applicable for the management of healthcare waste and current management practices in Ethiopia. Waste Manag Res.

[14] Pruss A, Giroult E, Rushbrook P (1999). Safe manangement of wastes from healthcare activities.

[15] Hill T, Lewicki P (2007). STATISTICS: Methods and Applications.

[16] IBM Corp (2013). Released 2013. IBM SPSS Statistics for Windows, Version 22.0.

[17] Sorensen G, Barbeau EM (2006). Integrating occupational health, safety and worksite health promotion: opportunities for research and practice. Med Lav.

[18] Chartier Y, Emmanuel J, Pieper U, Pruss A, Rushbrook P, Stringer R (2014). Safe management of wastes from health-care activities.

[19] Ministry of Environment: Hospital waste management rules (2005). SRO 1013 (1)/2005.

[20] Ferdowsi A, Ferdosi M, Mehrani Z, Narenjkar P (2012). Certain hospital waste management practices in isfahan, iran. Int J Prev Med.

[21] Brunot A, Thompson C (2010). Health care waste management of potentially infectious medical waste by healthcare professionals in a private medical practice: a study of practices. Sante Publique.

[22] Freedman AM, Simmons S, Lloyd LM, Redd TR, Alperin MM, Salek SS (2014). Public health training center evaluation: a framework for using logic training models to improve practice and educate the public health workforce. Health Promot Pract.

[23] Chethana T, Thapsey H, Gautham MS, Sreekantaiah P, Suryanarayana SP (2014). Situation analysis and issues in management of biomedical waste in select small health care facilities in a ward under Bruhat Bengaluru Mahanagara Palike, Bangalore. India J Community Health.

[24] Larson EL, Wong-McLoughlin J, Ferng YH (2009). Preferences among immigrant Hispanic women for written educational materials regarding upper respiratory infections. J Community Health.

[25] Arisanti N (2012). The effectiveness of face to face education using catharsis education action (CEA) method in improving the adherence of private general practitioners to national guideline on management of tuberculosis in Bandung. Indonesia Asia Pac Fam Med.

